# Human Platelet Lysate as a Xeno Free Alternative of Fetal Bovine Serum for the In Vitro Expansion of Human Mesenchymal Stromal Cells

**Published:** 2016-07-01

**Authors:** Saeed Mohammadi, Mohsen Nikbakht, Ashraf Malek Mohammadi, Mahdi Zahed Panah, Mohammad Reza Ostadali, Hajar Nasiri, Ardeshir Ghavamzadeh

**Affiliations:** 1 Hematology-Oncology and Stem Cell Transplantation Research Center, Tehran University of Medical Sciences, Tehran, Iran; 2 School of Allied Medical Sciences, Qazvin University of Medical Sciences, Qazvin, Iran

**Keywords:** Mesenchymal stromal cells, Umbilical cord blood, Platelet lysate, Immunomodulatory properties, Cell therapy

## Abstract

**Background:** Mesenchymal stromal cells (MSCs) are employed in various different clinical settings in order to modulate immune response. Human autologous and allogeneic supplements including platelet derivatives such as platelet lysate (PL), platelet-released factors (PRF) and serum are assessed in clinical studies to replace fetal bovine serum (FBS). The immunosuppressive activity and multi-potential characteristic of MSCs appear to be maintained when the cells are expanded in platelet derivatives.

**Materials and Methods:** Platelet-rich plasma was collected from umbrical cord blood (UCB). Platelet-derived growth factors obtained by freeze and thaw methods. CD62P expression was determined by flow cytometry. The concentration of PDGF-BB and PDGF-AB was detemined by ELISA. We tested the ability of a different concentration of PL-supplemented medium to support the ex vivo expansion of Wharton's jelly derived MSCs. We also investigated the biological/functional properties of expanded MSCs in presence of different concentration of PL. The conventional karyotyping was performed in order to study the chromosomal stability. The gene expression of Collagen I and II aggrecan and SOX-9 in the presence of different concentrations of PL was evaluated by Real-time PCR.

**Results:** We observed 5% and 10% PL, causing greater effects on proliferation of MSCs .These cells exhibited typical morphology, immunophenotype and differentiation capacity. The genetic stability of these derivative cells from Wharton's jelly was demonstrated by a normal karyotype. Furthermore, the results of Real-time PCR analysis showed that the expression of chondrocyte specific genes was higher in MSCs in the presence of 5% and 10% PL, compared with FBS supplement.

**Conclusions:** We demonstrated that PL could be used as an alternative safe source of growth factors for expansion of MSCs and also maintained similar growing potential and phenotype without any effect on chromosomal stability.

## Introduction

 Mesenchymal stem cells (MSCs) are multipotent cells that can differentiate into several types of cells. The first published report by Friedenstein et al. describing the expansion of an adherent, spindle-shaped population of cells from whole human bone marrow. ^1^^,^^2^  MSCs or MSC-like cells have also been expanded from other tissues including adipose tissue, umbilical cord blood (UCB), dental pulp, amniotic fluid, and numerous other sources. ^2^^,^^3^  Human MSCs (hMSCs) also have immunosuppressive and anti-inflammatory effects that might represent an attractive cell source for therapeutic applications.^4^ These cells dictate T cell functions which suppress the adaptive immune response and it was also revealed that MSCs inhibit dendritic cells maturation, enhance anti-inflammatory functions and decline the production of inflammatory cytokines.^   5 ^ For instance, immunosuppressive capacities of hMSCs facilitate and promote hematopoietic stem cell (HSC) engraftment. Furthermore, it has been also demonstrated that the use of these cells reversed severe acute GVHD.^   6 ^ Due to rare population within source, to achieve a minimum cell dose, ex vivo expansion is required. ^7^^-^^9^  Fetal bovine serum (FBS) can be used for mesenchymal expansion as a source of growth factors in the standard protocol for cellular therapy. ^10^^,^^11^  Recently, human autologous and allogeneic supplements including platelet derivatives, platelet lysate (PL) and platelet-released factors (PRF) and serum, are assessed in clinical studies to replace FBS. ^12^^,^^13^ 

The immunosuppressive activity of MSCs appears to be maintained when the cells are expanded in PL, ^11^^,^^14^  but it remains controversial.^        15 ^ The most important growth factors of platelets are platelet derived growth factor (PDGF) and transforming growth factor β (TGF-β).^        16 ^ Other growth factors releasing from the platelet granules are fibroblast growth factors (FGF-1 and FGF-2),^   17 ^ insulin-like growth factor-1 (IGF-1), ^18^^,^^19^  epidermal growth factor (EGF) and vascular endothelial growth factor (VEGF).^        20 ^ PDGF is a polypeptide and consists of two disulfide-bonded amino acid chains that bind with different affinities to two different but structurally related cell-surface receptors. Human platelets comprise all three isoforms, PDGF-AA, PDGF-AB and PDGF-BB. ^21^^,^^22^  Human platelet lysate (HPL) containing media was recently described as a possible substitute for FBS-containing media to expand of MSCs for therapeutic application.^        14 ^

Recently, various reports confirmed that effect of HPL on the ex vivo expansion of MSCs and also functional effects of MSCs expanded in PL and in FBS-containing media.^        23 ^ So, the aim of present study was to evaluate the efficacy of PL growth factors on expansion and differentiation of MSCs derived from Wharton’s jelly.

## MATERIALS AND METHODS

**Collection and preparation of Platelet Rich Plasma**

Platelet-Rich Plasma (PRP) was collected from three UCB unites and handed over to Shariati Hospital, Cord Blood Bank after obtaining signed and written informed consents. The PL was prepared according to method described by Bernardo et al.^11^ PRP was frozen at -80°C and subsequently, thawed at 37°C for three times to obtain the release of platelet-derived growth factors. Heparin (5000 UI) was added to the platelet bags to avoid gel formation. Products were centrifuged three times at 900 g for 30 min to remove platelet bodies. Finally, PL preparations obtained through this procedure were pooled in a single culture supplement for the generation and expansion of Wharton’s jelly-derived MSCs.

**Flow cytometric analysis of CD62P**

Platelet sediment was resuspended in HEPES buffer (Sigma-Aldrich, MO, USA) (200 μl) and incubated with saturating concentrations of FITC-anti-CD62 (BD Biosciences, CA, USA) at room temperature for 30 min in darkness. Subsequently, samples were incubated with a saturating concentration of PE-anti-CD41 antibody (BD Biosciences, CA, USA), which is used to set a gate for platelet events during the analysis. After incubation with labeled antibodies, samples were diluted in 1 ml of sodium citrate solution (3.8%) in Dulbecco’s phosphate buffered saline and centrifuged at 750 g for 5 min. Expression of CD62 was quantified as CD62 positive platelets (%) identified by binding of FITC-labeled CD62 antibody to the surface of platelets.

**Measurement of PDGF-AB and PDGF-BB**

A quantitative sandwich enzyme-linked immunosorbent assay (ELISA) (PDGF Human ELISA Kit Abcam, CA, USA) was used to examine the amount of PDGF-BB and PDGF-AB in PL supernatants. The immunoassays were performed following manufacturer′s instructions. Triplicate measurements were performed for all assays.

**Collection and selection of** **Wharton's jelly**

Wharton’s jelly stem cells were collected from full-term delivery. The Institutional Review Board of Tehran University of Medical Sciences approved the study.

**Isolation and culture of Wharton’s jelly derived MSCs**

MSCs were isolated from Wharton's jelly by enzyme digestion and density gradient centrifugation by Ficoll-Hypaqu (Stem Cell Technologies, Vancouver, Canada). Cells plated in non-coated 75 cm^2^ polystyrene culture flasks (Greiner bio-one, Kremsmünster, Austria) at a density of 160,000/cm^2^ in complete culture medium Mesencult, (Stem Cell Technologies, Vancouver, Canada) supplemented with 2 mM L-glutamine, 50 μg/mL gentamycin (Gibco, Carlsbad, CA) and 2-10% PL. Cultures were maintained at 37°C in a 5% CO2 humidified atmosphere. After 48h, non-adherent cells were discarded. Cultures were maintained at 37°C in a 5% CO2 humidified atmosphere. Culture medium was replaced twice a week. Upon the appearance of MSC-like clones, cells were harvested using trypsin (Gibco, Carlsbad, CA), replated for expansion at a density of 4000 cells/cm2 and propagated in culture until reaching a senescence phase. In order to reveal any change in morphology and/or proliferation rate, senescent cells were monitored up to 8 weeks. Cell growth was analyzed by direct cell counts and cumulative population doublings were determined. The number of population doublings was calculated using the formula log10 (N)/log10 (2), where N=cells harvested/cells seeded and results are expressed as cumulative population doublings.

**Immunophenotypic characterization of Wharton's jelly derived MSC**

Fluorescein isothiocyanate (FITC) and phycoerythrin (PE) labeled monoclonal antibodies specific for the following antigens were employed: CD45, CD14, CD34, CD90, CD73, CD105 (DAKO, Glostrup Municipality, Denmark) for the assessment of the surface phenotype of the MSC by a Partec PAS III instrument (Münster,Germany) using Flow Max version 2.5.

**Karyotyping**

Cytogenetic analysis was performed on three Wharton’s jelly derived MSC by conventional karyotyping.

**Chondrogenic differentiation of** **hMSCs**

After three passages, hMSCs were obtained and seeded at cell density 1×10^6^ cells/ml. Then, the cells were incubated in chondrogenic medium consisting of DMEM-High Glucose (Gibco, Carlsbad, CA) supplemented with 50 mg/ml bovine serum albumin (Sigma-Aldrich, MO, USA), 5 µg/ml ascorbate-2-phosphate (Sigma-Aldrich, MO, USA), 1% insulin-transferrin-selenium (Sigma-Aldrich, MO, USA), 1 nM dexamethasone (Sigma-Aldrich, MO, USA), 5 µg/ml linoleic acid (Sigma-Aldrich, MO, USA ), 1% penicillin-streptomycin (Gibco, Carlsbad, CA) and 10 ng/ml TGF- β1 (Sigma-Aldrich, MO, USA) and different concentration of PRP from 5-10% for 2 weeks. The culture medium was changed every 2-3 days.

**Cell proliferation assays**

3-(4, 5-dimethylthiazol-2-yl)-2, 5 diphenyltetrazolium bromide (MTT) (Sigma-Aldrich, MO, USA) assay was used for the quantitative determination of cellular proliferation. The confluent second-passage cells were trypsinized and re-suspended. The cells were counted and seeded at a density of 2000 cells per well in 96-well plates. The cells were cultured for 24 hours.

After the 24 hours adhesion period, the medium was removed and replaced with DMEM (Gibco, Carlsbad, CA) in combination of 10% FBS (negative control), 2-10% PL supernatants (positive control), respectively.

Medium was replaced every 3 days. MTT was assayed at day 2-6 after treating to establish a growth curve of cells cultivated. The cells were incubated with 5 mg/ml of MTT in the last 4 hours of culture period tested. The medium was removed and formazan salts were dissolved with 150 μl of dimethylsulphoxide (Sigma-Aldrich, MO, USA) and the absorbance was determined at 570 nm/630 nm with an ELISA reader. Each experiment was repeated 3 times for each group.

**RNA isolation**

Expression rate of collagen type I and II, SOX-9 and aggrecan was evaluated 14 days after chondrogenic differentiation. Total RNA was extracted by using the TRI reagent (Sigma-Aldrich, MO, USA) according to the manufacturer's instructions. The RNA pellets were reconstituted in DEPC (Sigma-Aldrich, MO, USA) treated water.

**Real-time PCR**

Complementary DNAs (cDNAs) were reverse transcribed from 1-2 µg of total RNA by use of cDNA synthesis kit (Fermentas Life Science, Vilnius, Lithuania) according to the manufacturer‘s instructions. Real-time PCR was performed by Step One Plus instrument (Applied Biosystem, CA, USA) using CYBER Green Quantitative RT-PCR kit (Takara Bio Inc, Shiga, Japan).

Primers were manufactured by Takapouzist (Tehran, Iran) (Table 1) and reactions were optimized and processed according to the manufacturer with initial denaturation/DNA polymerase activation at 95°C for 15 min followed by PCR: 95°C for 15s, variable annealing temperature for 5s and 59°C for 30s repeated for 40 cycles. Glyceraldehyde 3-phosphate dehydrogenase (GAPDH) was used as a housekeeping gene. Data were calculated as relative expressions according to the 2^−ΔΔCT^ principle.

**Statistical analysis**

SPSS 18 was used to perform statistical analysis. The significance of differences between experimental variables was determined by the use of two tailed student's test in order to make a comparison between the expanded group with PL and control group. All experiments were performed in triplicate and results were expressed as the mean ± standard deviation (SE). Statistical significance was definedat *p<0.05, **p<0.01 and ***p<0.001, compared to corresponding control.

## Results

**CD62P expression as a suitable surrogate of PDGF release from Platelets**

To determine platelet degranulation and PDGF release from platelets, α granules and CD62P were determined in all samples that were frozen and thawed.

**Table 1:** Nucleotide sequences of primers used for Real-time PCR

**Primer name**	**Sequences (5'->3')**
Type I Collagen-Forward	TTGTACAGACATGACAAGAGGC
Type I Collagen-Reverse	CTCTACCTGGGTACTACCCA
Aggrecan-Forward	CAGAGTGAAATCCACCAAGT
Aggrecan-Reverse	TGTCCGTGGACAAACAGGTA
SOX-9-Forward	TACGACTACACGCACCACCA
SOX-9-Reverse	TTAGGATCATCTGCGCCATC
Type II Collagen-Forward	ACACAGCGCCTTGAGAAGAG
Type II Collagen-Reverse	TTCTACGGTCTCCCCAGAGA

**Figure 1:**
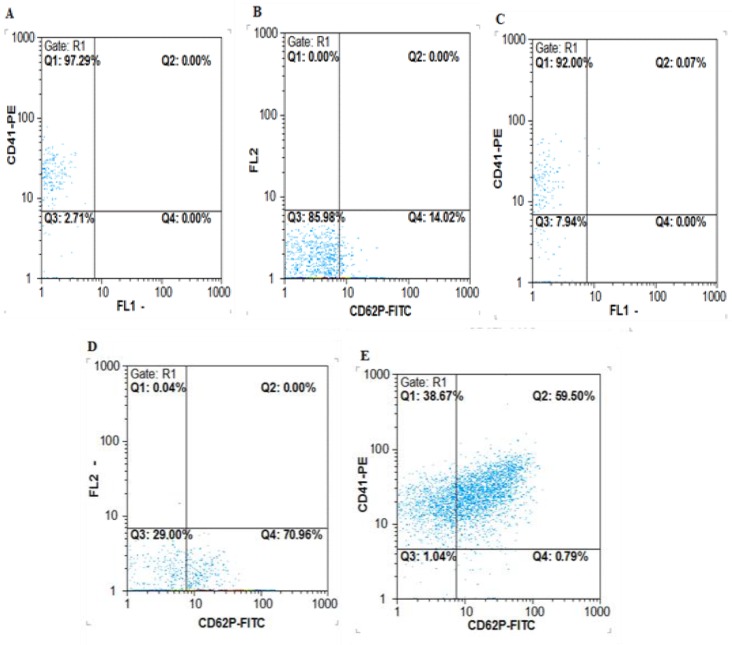
Quantification of CD62P expression on CD62 positive platelets. (A): CD41 in fresh sample (B): CD62P in fresh sample (C): CD41 gate control in freeze and thaw platelet samples. (D): CD62P expression on freeze and thaw samles. (E): CD41/CD62P control in freeze and thaw platelet sample

The expression level of CD62P quantified as CD62P positive (%) platelets showed that 70.96% of platelets release their PDGF by freeze-thaw method. Results have shown in Figure 1 (A-E).

**Quantification of PDGF in PL with ELISA assay**

**Figure 2:**
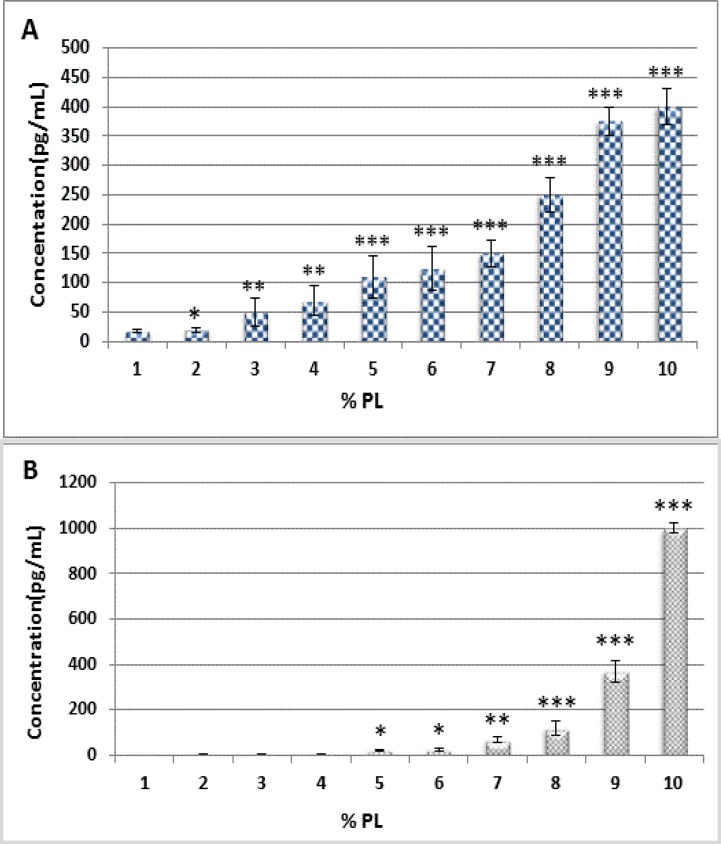
Quantification of cytokine molecules (pg/mL ) in adult platelet-lysate (PL). (A): PDGF-AB, (B): PDGF-BB. Values are given as mean ± SE of three independent experiments. Statistical significance were defined at *p<0.05, **p<0.01 and ***p<0.001 compared to corresponding control

The concentrations of PDGF-BB and AB isoform in HPL are illustrated in Figure 2 (A-B).

**Immunophenotypic characterization of MSCs**

Immunophenotyping was carried out using a panel of antibodies to a number of surface antigens routinely used for the characterization of MSCs. Wharton’s jelly MSCs were analyzed at the end of (P-V) from culture initiation and characteristic immunophenotypes have shown in Table 2. They were negative for hematopoietic markers CD34, CD45 and CD14. Additionally, all Wharton’s jelly derived MSCs were found to be positive for matrix receptors CD44, CD105, CD90, CD73 and CD166.

**Morphological and histological characterization of UBC-derived MSCs**

Wharton’s jelly at full-term delivery was selected according to the ‘quality’ criteria described in the materials and methods section. Nucleated cells were separated and cells were seeded in MSCs at a density of 1.0 × 106/cm2 cultured in medium supplemented with 1-10% PL. Adherent cells with fibroblastic morphology could be observed as early as 48 hours.

**Table 2:** Proliferation of mesenchymal stem cells in the presence of platelet lysate with different concentration of platelet factors. P1: Passage 1, P2: Passage 2, PL: Platelet Lysate

**PL%**	**PDGF-** **BB** **pg/ml**	**PDGF-** **AB** **pg/ml**	**P1**	**P2**	**Fold** **Change **%
2	979.5 ± 21.85	432.33 ± 31.08	0.176 ± 0.007 × 10^6^	1.5 ± 0.2 × 10^6^	85.2
5	80.04 ± 11.15	172.33 ± 22	0.319 ± 0.005 × 10^6^	5.56 ± 0.25 × 10^6^	175
7	25.88 ± 4.44	145.33 ± 35.5	0.215 ± 0.01 × 10^6^	2.56 ± 0.25 × 10^6^	120
10	2.64 ± 0.93	24.09 ± 3.82	0.283 ± 0.009 × 10^6^	5.5 ± 0.1 × 10^6^	196

**Figure 3:**
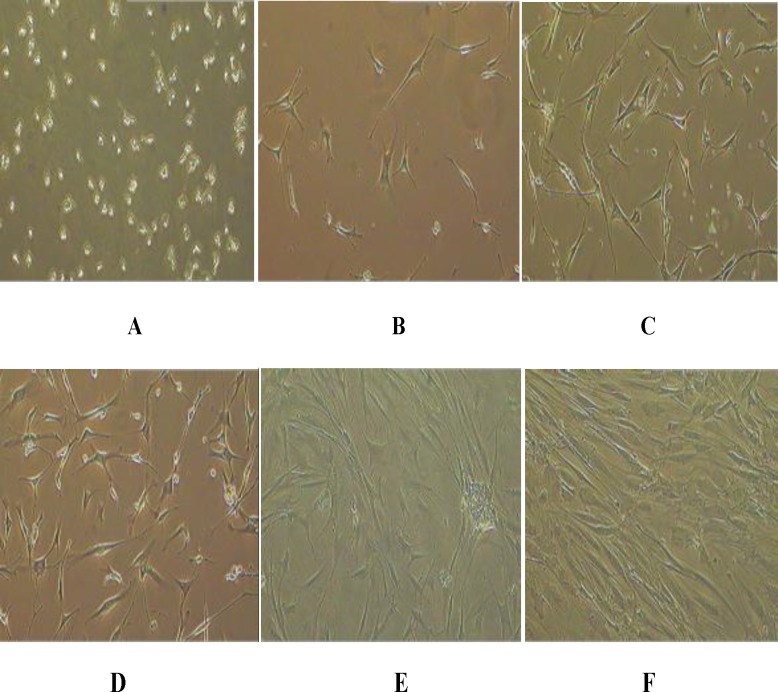
Culture-expanded human mesenchymal stem cells exhibit spindle-shaped fibroblastic morphology following culture expansion ex vivo

The cells formed a monolayer of homogenous bipolar spindle-like cells with a whirlpool-like array within 2 weeks (Figure 3: A-F). The cultures at P0 were monitored up to 4 weeks to allow identification of Wharton’s jelly-derived MSCs which gave rise to MSC-like clones. MSC clones were expanded ex vivo and characterized for their morphology, differentiation potential and proliferative capacity.

Wharton’s jelly-MSCs were induced to differentiate into osteoblasts, adipocytes and chondrocyte to examine this capacity by histological staining. The cells were able to differentiate into osteoblasts, as demonstrated by the histological detection of alkaline phosphatase activity (purple reaction) and calcium deposition stained with Alzarin Red (Figure 3: A) and into adipocytes, as revealed by the formation of lipid droplets, stained with Oil Red O (Figure 2: B). Cumulative cell counts from P0 to P4 for Wharton’s jelly-MSCs cultured in the presence of 1-10% PL average cell counts obtained were demonstrated in Table 3. The median time to reach 80% confluence for all passages (P1 to P4) was 12 days. Culture in PL-containing media promotes strong MSCs proliferation. The total cumulative number of MSCs expanded in 10% FBS (FBS-MSCs) was significantly lower than those obtained upon expansion in PL-containing media (PL-MSCs).

Indeed, there was a much higher proliferation rate in PL-MSCs that was statistically significant in all passages. The difference increased with each passage (Figure 3: F).

**Table 3:** Flow cytometric analysis of expanded hMSC: Analysis of positive and negative human cord blood derived MSC surface antigen by flow cytometry for cells expanded in SCM and SCM + PL. SCM: serum containing media; PL: platelet lysate

**Flow cytometry**	**SCM**	**PL** **+** ** SCM**
Target CD14 CD34 CD45	Negative Human MSC (%) 8.83 6.77 3.42	8.21 6.67 3.3
Positive Human MSC (%)		
CD44 CD73 CD90 CD105 CD166	87.55 98.51 98.78 37.32 96.88	87.56 98.72 98.01 37.04 97.3

**Cytogenetic analysis**

**Figure 4:**
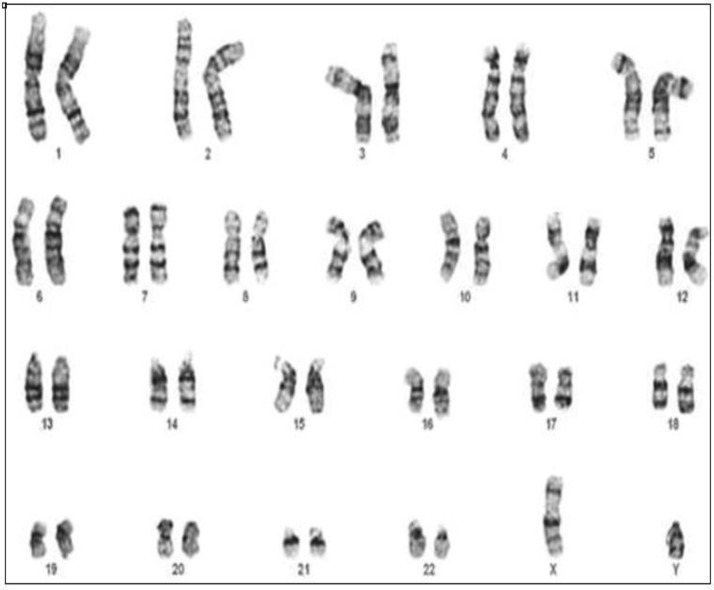
Mesenchymal stem cells at Passage 4. Normal complete karyogram (46,XY)

Cytogenetic analysis was performed on three Wharton's jelly MSCs at three different passages, (2-4). At P4, cultured cells had previously undergone cumulative population doublings. In spite of massive expansion, no structural abnormalities were detected (Figure 4).

**Differentiation studies**

**Figure 5:**
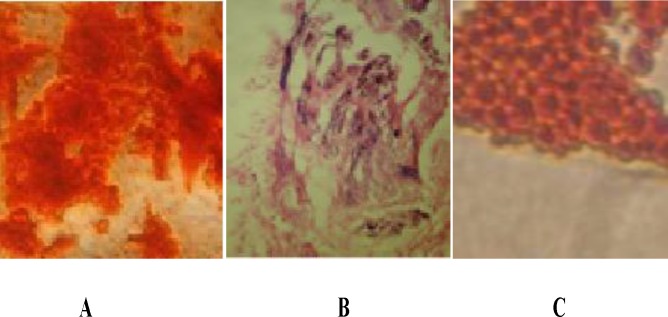
Under appropriate inducing conditions, the culture will demonstrate adipogenic differentiation, chondrogenic and osteogenic. **(A):** Alizarin Red S staining **(osteogenesis), (B):** Hematoxylin and Eosin staining **(chondroeogesis), (C):** Oil Red O staining **(adipogenesis)**

MSCs started to generate adipocyte, osteocyte and chondrocyte after culture with PL and differentiation media which was established by staining. (Figure 5: A-C)

**PL induces MSCs proliferation**

**Figure 6:**
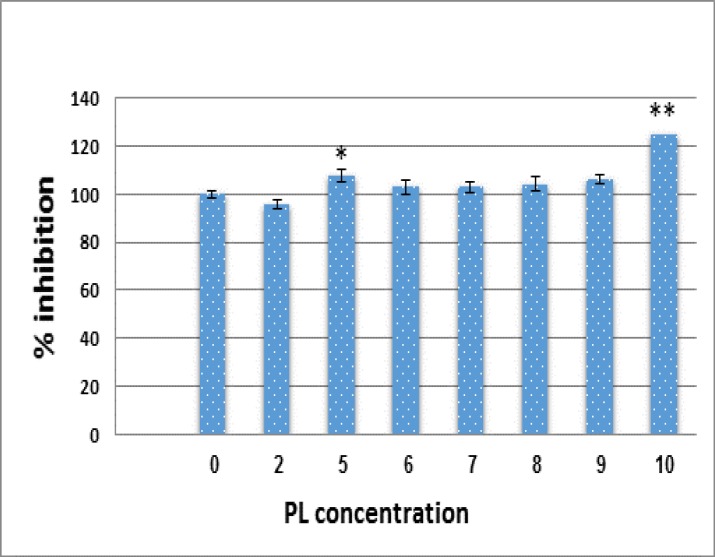
Effects of PL on cell viability and prolifferation. Growth effect of PL in different concentration (2-10%) was measured by MTT assay following 2-6 days exposure. Values are given as mean ± SE of three independent experiments. Statistical significance were defined at *p<0.05, **p<0.01 and ***p<0.001 compared to corresponding control

Initial studies were performed to elucidate the prolifration effects of PL on MSCs. After treatment of these cells with different concentrations of PL for 2-6 day, growth suppressive and proliferative effect

were assessed by MTT methods. Results showed that PL induced cell proliferation in MSCs in both dose- and time-dependent manners (Figure 6).

**PL induces up-regulation of SOX-9 and collagen type II**

**Figure 7:**
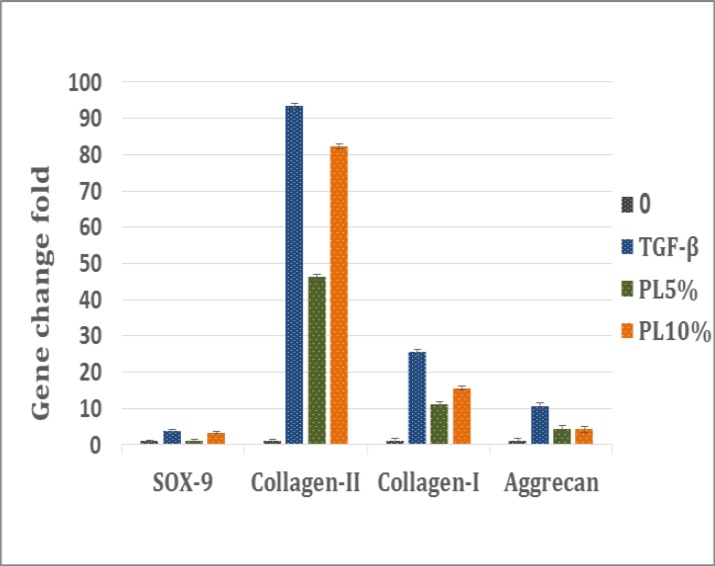
Effects of PL (5 and 10 %) and TGF-β treatment on SOX-9/Collagen II/Collagen I/Aggrecan genes expression by Real-time PCR in hMSCs in comparison to control group. Values are given as mean ± SE of three independent experiments

The general patterns of gene expression are summarized in Figure 7. The mRNA expression of collagen type I, collagen type II, SOX-9 and aggrecan in differentiated cells was evaluated after 14 days. No significant difference in the expression of aggrecan mRNA was observed.

The mRNA level of SOX-9 and collagen type II gene was expressed significantly different in TGF beta group as compared to PL group. The SOX-9 mRNA expression noticeably upregulated in 10% PL group. In terms of the SOX-9 expression, there was a significant alteration in different concentration of PL. About collagen type I, the expression level was significantly different in TGF beta. The mRNA level of collagen type II enhanced significantly in 5% and 10% PL groups.

## Discussion

 The use of FBS during in vitro culture expansion of MSCs might pose a potential hazard due to proteins and porcine macromolecules. The internalization of these macromolecules in stem cells can transmit viral/prion disease. Moreover, these molecules serve as antigenic substrates on transplanted cells and cause immunological reactions.

FBS is not desirable due to safety and other concerns for clinical application. In some research studies, anaphylaxis and other allergic reactions have been defined in the patients transplanted with the cells supplemented with FBS. In the culture medium proposed, FBS could be replaced with human AB serum or PL and can be considered as better choice for clinical application. The platelets are known as a source of mitogenic growth factors and molecules that promote tissue repair and angiogenesis. However, preparation of PL is highly variable and although this human source is precisely screened for potential infections, the risks of transmitted disease by human source products still need to be considered. Autologous HPL could be considered as a safe source for MSCs expansion^        13 ^ but using HPL may cause major problems in patients because of their illness.

Some researchers prepared a plasma-free PL in human albumin (HA) solution, for in vitro expansion of MSCs.   ^13^^,^^24^  These researchers demonstrated that MSCs expanded in PL-HA-supplemented medium proliferated more expansively than those supplanted with FBS.^        24 ^

The immune phenotype property of MSCs cultivated in FBS needs further investigation. Recent work comparing serum sources shows that the use of PRP results in a significantly higher expansion rate compared with FBS containing medium.^        24 ^

The freeze-thaw process has been well defined for PL production. The freeze/thaw cycle usually is repeated two to three times but the efficacy of the growth factor release is related to the disruption of the platelet and the granulations.^        10 ^ In the present study, the attempt was made to evaluate the effect of different concentration of PL containing the known growth factors and chemokine as an important and safe growth promoting factors on MSCs expansion compared to FBS containing media.

PL can be obtained either from autologous or allogeneic source. The allogeneic platelets could be obtained from cord blood, random platelet or by apheresis.^        25 ^ Similar to our study, Iudicone et al. used the allogeneic PL obtained by combining 2-6 units' platelet pools in additive solution (AS) for MSCs expansion. Avanzini et al. used the autologous platelets by apheresis method for their research.^        25 ^ In the present study, PRP was obtained from UCB units. The CD62P expression as an activation marker on the surface of platelets was considered as an indicator of platelet degranulation and subsequently secretion of granular content. The CD62P expression on the surface of platelet reflected the secretion of PDGF as a major ingredient of granules and could be stimulated by freeze and thaw of platelets. The flow cytometric detection of CD62P expressed on the platelet surface after freeze and thaw of platelets followed by degranulation is a standard method used to measure the platelet activation in different reports of research works.   ^11^^,^^26^  The percentage of CD62P positive platelets in the total platelet population (%) is commonly used for describing the platelets activation. In good agreement with our study, Michelson et al. reported that the percentage of CD62P positive platelets could be considered as the proportion of activated platelets but not related to the quantity of surface CD62P antigen expressed by granulated platelet.^        27 ^ On the other hand, Evangelista et al. showed that MFI constitutes the mean epitope density of CD62P molecules on the average platelet surface and reflects the activity of the single platelet but not its quantity.^   26 ^

Three of the most important growth factors which can play on proliferation and differentiation of MSCs are PDGF-BB, bFGF, and TGF-β1. Although PDGF-BB and bFGF had positive effects on cell growth individually, TGF-β1 appeared to provide no enhancement of cell proliferation on its own. Interestingly, although combinations of any two growth factors appeared to provide minimal or no significant augmentation of cell proliferation compared with single factors, the combination of all three factors provided a significant synergistic effect. HPL, which is rich in PDGF, has been used to replace FBS for MSCs expansion.^        12 ^ PDGF is one of the crucial components which mediate the proliferation of vascular smooth muscle cell and also migration which may happen in initial stage of hyperplasia in the process of restenosis and atherosclerosis. Platelet granules are containing mainly PDGF-AB and PDGF-BB.   ^21^^,^^22^^,^^28^  The content of PDGF-AB, TGF-β1 and bFGF in PL may account for its efficacy in promoting MSCs growth.^        10 ^ Accordingly, the growth activating potency of PDGF-BB is reported to be about 4-fold larger than that reported for PDGF-AB.^22^ As already described by others, we confirmed that PL mainly contains large amounts of growth factors designated to play a role in both human MSCs proliferation and differentiation but the concentrations of these growth factors reported by the various groups were different.   ^29^^,^^30^  We have revealed that PDGF-AB/BB are important and essential growth factors for the proliferation of MSCs as described by Nedeaue et al.^        31 ^ Of note, the neutralizing antibodies were not able to abolish the growth-promoting effect of PL completely.

Therefore, we hypothesize that in addition to the essential components PDGF-AB/BB, other constituents of PL are important for their full biologic activity. Although this study is focused on the growth-promoting effects of PL on MSCs, the other various constituents of PL might also be important for proliferation, differentiation   ^32^^-^^34^  and migration of MSCs.   ^35^^,^^36^  The PRP was derived from cord blood forced to degranulation by freeze and thaw to release the growth factors. Our analysis revealed that equivalent concentration of platelets generates almost the same proportion of growth factors. Our data showed degranulated platelets significantly released the higher levels of PDGF-AB/BB growth factors which have been repeatedly reported that has significant effects on the proliferation of MSCs.^        37 ^ Our results also give further prove of evidence for the importance of PDGF signaling in growth and proliferation of MSC reported by many research groups. Recent work comparing growth factors sources shows that the use of PL results in a significantly higher expansion rate compared with FBS-containing media. PL exhibited an enhanced proliferative ability without compromising their differentiation capacity or the immune phenotype. To this end, it could also be shown that PL-cultured MSCs have immunomodulatory capacities compared with their FBS-cultured counterparts including a beneficial inhibitory effect on immune cell proliferation and an unaffected viral T-cell immunity.   ^38^^-^^40^  Further experimental works are required to identify important components of PL and their effects on MSCs biology. In present study, Real-time PCR result revealed that the expression collagen type II and aggrecan were significantly increased as compared to the control group (p<0.05). Based on the results of our study, it seems that PL as source of growth factor may lead to a better induction of chondrogenesis.

Evidence show that chondrogenesis is recognized by an increase in expression and accumulation of collagen Type II and aggreacan genes compared to collagen Type I and SOX-9. ^41^^,^^42^  These results indicate that the ratio of chondrogenic-related genes expression is increased several fold. Consequently, differentiation media containing 5 and 10% of PL had a lower expression of collagen Type I, higher expression of collagen Type II and aggrecan, when compared to MSCs expanded with FBS.^        43 ^

Results of our study showed that PL can be considered as an appropriate source of growth factor for differentiation of MSCs due to higher expression of the genes involved in chondrocyte differentiation compared to FBS.^        44 ^ In this study, no clonal chromosomal aberrations were identified in any of the analyzed cases. However, increases in genetic instability probably depend on the time of cultivation has been reported by Binato et al.^        45 ^ Also several signs of chromosomal instability were observed including chromatid gaps, chromosomal breaks and tetraploid metaphases by Borgonovo et al.^   46 ^ These abnormal mitotic mechanisms are described in the progression of malignant mesenchymal tumors, where the increased frequency of chromosomal aberrations can be explained by a process initiated by telomere dysfunction and an aphasic bridges.^   47 ^

## CONCLUSION

 our findings show that PL might be as a good and safe replacement for FBS in MSCs expansion without any clonal chromosomal aberrations in expanded cells.
